# Effects of progressive multimodal resistance training with varied muscle actions and range of motion on bone mineral density in osteopenic older women: a pilot study

**DOI:** 10.3389/fphys.2026.1811518

**Published:** 2026-07-06

**Authors:** Albeiro Dávila-Grisalez, Yovanny A. Tascón-Martínez, Jhony A. Rodríguez-Gómez, Juan C. Calderón-González, Diana M. Bedoya-Salazar, Diego A. Bonilla, César A. Mazuera-Quiceno

**Affiliations:** 1 Physical Education, Recreation and Sports Program, Universidad Central del Valle del Cauca, Tuluá, Colombia; 2 Research Group in Physical Culture, Motor Sciences and Health (GICMOS), Universidad Central del Valle del Cauca (UCEVA), Tuluá, Colombia; 3 Research Group in Integrative Physiology (DBSS-Phi), Dynamical Business and Science Society – DBSS International SAS, Bogotá, Colombia; 4 Hologenomiks Research Group, Department of Genetics, Physical Anthropology and Animal Physiology, University of the Basque Country (UPV/EHU), Leioa, Spain

**Keywords:** bone mineral density, multimodal resistance training, older women, osteopenia, pilot study

## Abstract

**Background:**

Resistance training improves bone health in patients with osteopenia, but high-load programs may not be ideal for everyone. This pilot study examined the effect of a progressive multimodal resistance training program on bone mineral density (BMD) in osteopenic women.

**Methods:**

Twenty older women (65.3 [3.23] years; 1.58 [0.08] m; 69.3 [12.9] kg) diagnosed with osteopenia were non-randomly assigned to experimental (*n* = 10) or control (*n* = 10) groups. After obtaining ethics approval and informed consent, experimental participants completed a 16-week multimodal resistance training program with varying muscle actions and range of motion (ROM). Lumbar spine and femoral neck BMD were assessed pre- and post-intervention by DXA. Estimation, robust, and Bayesian statistics were employed.

**Results:**

The experimental group showed BMD increases at the lumbar spine (Δ=+0.05 (0.04) [95% CI: 0.03, 0.08] g·cm^-2^; d_unb_=0.20; p=0.026) and the femoral neck (Δ=+0.05 (0.03) [95% CI: 0.02, 0.07] g·cm^-2^; d_unb_=0.35; p=0.003), exceeding the predefined MCID threshold (+0.03 g·cm^-2^). No changes were found in control. Between-group comparisons favored the experimental group (lumbar: p=0.003; femoral: p=0.010). Bayesian ANCOVA provided strong support treatment effects (BF_m_>10^7^; P(M|data)>0.96), consistent with frequentist ANCOVA findings (η²p>0.48).

**Conclusion:**

This non-randomized pilot intervention study found preliminary evidence that multimodal resistance training improved BMD in osteopenic older women. Findings are limited by sample size and support a future definitive trial.

## 1 Introduction

Aging represents an intrinsic biological process characterized by progressive declines in organ system function, including deterioration of the musculoskeletal system (Cannataro et al., 2021; Liao et al., 2021). Women may lose up to 40% of their bone mass beginning as early as their third decade of life (Hirschfeld et al., 2017; Azzolino et al., 2021). However, this bone loss does not occur uniformly and is significantly influenced by multiple factors. Age-related declines in bone repair capacity are driven by three primary mechanisms, including osteocyte apoptosis, reduced osteocyte density, and the accumulation of microdamage (Calvi, 2019; Weaver and Peacock, 2019; Yao et al., 2024). These pathological processes are further exacerbated by mineralization of the lacunar spaces (micropetrosis), which impairs canalicular fluid flow and microdamage detection, ultimately compromising bone regenerative capacity (Jilka and O’Brien, 2016; Hemmatian et al., 2017; Milovanovic and Busse, 2023). Additionally, estrogen deficiency may accelerate bone loss through mechanisms distinct from those associated with aging (Padilla Colón et al., 2018; El Miedany, 2022).

Osteoporosis and osteopenia represent a substantial global public health burden, affecting millions of individuals worldwide (Salari et al., 2021). Current estimates indicate that osteoporosis affects 19.7% of the global population, whereas osteopenia shows a higher prevalence, reaching 40.4%. In developed countries, particularly concerning patterns have been observed, with osteopenia rates (22.1%) exceeding the prevalence of osteoporosis (14.5%) (Xiao et al., 2022). In the United States, 19.6% of women over the age of 50 have osteoporosis, while 51.5% present with osteopenia (Sarafrazi et al., 2021). Data from Latin America remain limited; however, emerging research reveals a significant regional prevalence: 50% of Argentinian women over 50 exhibit osteopenia; Mexican women show a 59% reduction in bone mass at the spine and hips; and Brazilian fracture rates suggest an osteopenia prevalence between 10.5% and 17% (Clark et al., 2024). In Colombia, a marked increase has been observed over the past decade, with prevalence rates of 34.46% for osteoporosis and 45.14% for osteopenia among women aged ≥60 years (Espitia-De-La-Hoz, 2021). Postmenopausal women exhibit particularly high susceptibility, with an osteopenia prevalence of 48.55% compared with their premenopausal counterparts (Fan et al., 2024).

Although resistance training significantly improves bone mineral density (BMD) in older adults (Watson et al., 2018; O’Bryan et al., 2022), the risk of injury and delayed-onset muscle soreness (DOMS) may represent important limitations, particularly when exacerbated by age-related declines in recovery capacity (Sousa et al., 2014). This underscores the need for carefully tailored programs that consider the physiological constraints of the geriatric population (Ciolac and Rodrigues-da-Silva, 2016; Collado-Mateo et al., 2021; Cannataro et al., 2022). In this context, contemporary research has explored a variety of training modalities aimed at improving BMD, including weight resistance training, elastic bands, whole-body vibration, and aquatic exercises, as part of combined strength and cardiorespiratory exercise programs in older women (Moreira et al., 2014; Nicholson et al., 2015; Borba-Pinheiro et al., 2016; ElDeeb and Abdel-Aziem, 2020; Ghosal and Bandyopadhyay, 2020; Banitalebi et al., 2021). These interventions systematically manipulate classical exercise prescription variables such as intensity (expressed as %1RM or 10RM), volume, duration, frequency, density, progressive overload, vibration parameters, postural complexity, and perceived exertion (Moreira et al., 2014; Nicholson et al., 2015; Borba-Pinheiro et al., 2016; de Oliveira et al., 2019; ElDeeb and Abdel-Aziem, 2020; Ghosal and Bandyopadhyay, 2020; Banitalebi et al., 2021).

Despite the availability of guidelines and multiple methods for controlling training intensity in older adults, the potential of muscle action type (i.e., concentric, eccentric, and isometric actions), as well as their manipulation in terms of sequence, duration, and combination during exercise execution, and range of motion (ROM) as prescriptive variables remains largely unexplored. These variables may offer safer and more adaptable alternatives to high-load protocols, particularly in older women with osteopenia, who are at increased risk of injury and exhibit lower tolerance to mechanical stress. Therefore, this non-randomized pilot study was designed to preliminarily evaluate the potential effectiveness, safety, and feasibility of a 16-week progressive multimodal resistance training program that systematically manipulates muscle actions and ROM. Given its pilot nature, this intervention period was selected to identify preliminary signals of effect and to generate effect size estimates to inform the design of a future large-scale definitive trial aimed at improving bone health in this population.

## 2 Materials and methods

### 2.1 Trial design

**Figure 1:**
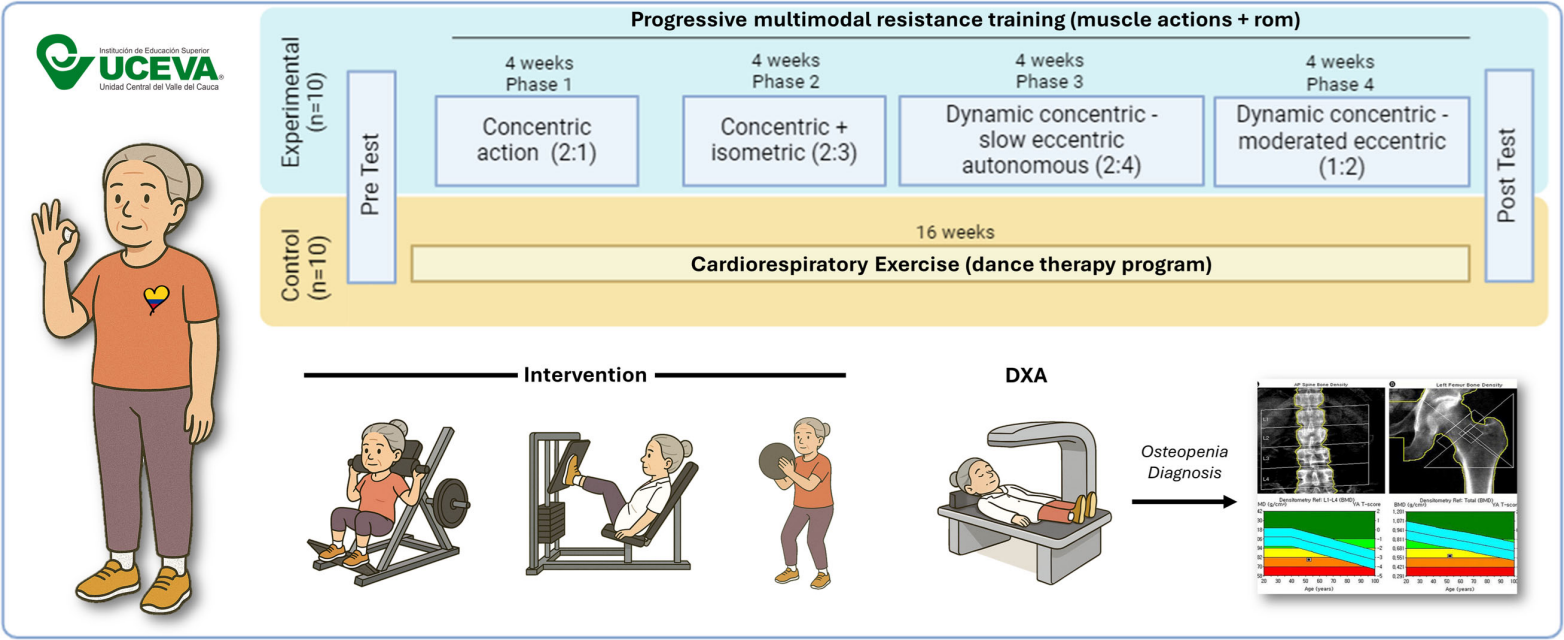
Experimental design of the study. DXA: Dual-energy X-ray Absorptiometry.

This was a two-arm repeated-measures non-randomized pilot study conducted in osteopenic older women. The study is reported in accordance with the Consolidated Standards of Reporting Trials (CONSORT) extension to pilot and feasibility trials (Eldridge et al., 2016). The study protocol was prospectively registered and publicly archived on Zenodo prior to the initiation of participant recruitment (DOI: 10.5281/zenodo.18487882), in order to ensure transparency and traceability of the study design. We evaluated the effects of a 16-week progressive multimodal resistance training program involving varied muscle actions and ROM on BMD in Colombian women diagnosed with osteopenia (**Figure 1**).

### 2.2 Participants

Older women from a municipality in southwestern Colombia (San Pedro, Valle del Cauca) were recruited for this pilot trial, conducted as part of an undergraduate thesis project in a physical education program at a public university. Participants were identified through community outreach efforts and informational sessions held at local health centers. Eligible participants were aged 60–70 years, had a confirmed diagnosis of osteopenia via dual-energy X-ray absorptiometry (DXA) (T-score between -1.0 and -2.5), and had not participated in a structured training program in the preceding six months.

Exclusion criteria included: i) osteoporosis (T-score < -2.5); advanced cardiovascular disease (heart failure class II-IV [NYHA], recent myocardial infarction [<6 months], unstable angina); severe renal failure (eGFR < 30 mL/min/1.73 m²); alcohol, psychoactive substance, or tobacco addiction; implants or foreign devices in the measurement area; active malignancy requiring treatment; endocrine or metabolic disorders (type 1/2 diabetes, thyroid/parathyroid disease, Paget’s disease of bone); recent (<1 month) barium exam or contrast injection for CT/radioisotope studies; use of medications affecting bone metabolism (bisphosphonates, SERMs, HRT, corticosteroids, aromatase inhibitors, anticonvulsants, anticoagulants, antiresorptives, bone-forming agents, anabolic steroids, or androgens).

Exit criteria were participant withdrawal, adverse effects preventing continuation, repeated absenteeism, development of exclusionary medical conditions, or medication changes. All older women were thoroughly informed about the research objectives, as well as the associated risks and benefits. Subsequently, the women signed an informed consent form in accordance with the ethical guidelines outlined in the Declaration of Helsinki (WMA - The World Medical Association, ).

### 2.3 Setting

The study was conducted at a university-affiliated sports science and physical activity center, equipped with strength-training machines, exercise tools (dumbbells, benches, medicine balls, lightweight discs), and supervised by professionals in sports science, medicine, and physiotherapy. A non-randomized pilot design was selected due to logistical and feasibility (budget) constraints, including limited sample availability and resource allocation.

### 2.4 Intervention procedures

The 16-week exercise intervention included multi-joint machine exercises (hack squat [Pure Hack Squat, Technogym, Cesena, Italy] and horizontal leg press [Pro2 Seated Leg Press, Life Fitness, Chicago, IL, USA]) alongside functional free-weight exercises designed to simulate activities of daily living.

The progressive multimodal resistance training program with varied muscle actions and ROM was implemented three times per week (non-consecutive days with four participants per session) in four phases: i) *Phase 1 (Weeks 1–4)*: Concentric (2 s) and assisted eccentric (1 s) actions (velocity ratio [VE] 2:1) on hack squat and leg press machine exercises. Horizontal training method (all sets of one exercise completed before progression); ii) *Phase 2 (Weeks 5–8)*: Concentric-isometric actions (VE 2:3); eccentric actions excluded for initial progression (LaStayo et al., 2014); iii) *Phase 3 (Weeks 9–12)*: Autonomous eccentric actions (VE 2:4); and, iv) *Phase 4 (Weeks 13–16):* Double sets with moderate ROM and vertical training (alternating exercises without rest; VE 1:2).

Range of motion (ROM) was standardized using a digital goniometer (Baseline 12–1027 Absolute Axis 360 Degree, Dedham, MS, USA). For the machine-based exercises, three ROM categories were defined: very limited (ROM #1), limited (ROM #2), and moderate (ROM #3). Very limited ROM (#1) was set between 120°–110° at the tibiofemoral joint and 125°–100° at the coxofemoral joint, with the aim of enhancing spinal protection. Limited ROM (#2) corresponded to ranges of 109°–100° (tibiofemoral) and 99°–80° (coxofemoral), while moderate ROM (#3) was defined between 99°–90° (tibiofemoral) and 79°–60° (coxofemoral). These ROM categories (#1–#3) were applied progressively across the intervention phases and are described in detail in **Supplementary Material 1**. The program also incorporated functional exercises simulating activities of daily living, such as chair stands, object retrieval, and step climbing, using adjustable dumbbells (5–12 lbs), low-height benches (10–30 cm), and medicine balls (2–10 lbs). Exercise complexity progressed from single-joint to multi-joint movements.

Exercises simulating activities of daily living used adjustable dumbbells (Bowflex, 5–12 lbs), low-height benches (10–30 cm), medicine balls (TRX, 2–10 lbs), and lightweight discs (Eleiko, 5–10 lbs). These exercises replicated fundamental and instrumental activities of daily living, including chair stands, object manipulation (carrying, retrieving, reaching), and step negotiation (ascending/descending). Exercise progression followed two dimensions; complexity (advancing from single-joint to multi-joint movements) and difficulty (increased via greater loads or elevated bench heights). A detailed week-by-week progression of the multimodal resistance training protocol is provided in **Supplementary Material 1**.

Baseline performance was established using unloaded machine exercises (platform weight = 44 lbs for hack squat and leg press), with emphasis on proper technique, individualized ROM parameters, and controlled muscle action durations (concentric, eccentric, isometric). Participants logged exercises and difficulties post-session. Attendance and protocol adherence were tracked. Motivational strategies included goal setting, weekly feedback, and monthly interviews. The control condition consisted of a structured and supervised dance therapy program (3 sessions/week), comprising a 10-minute warm-up (low-intensity walking and joint mobility exercises for the upper and lower limbs), followed by 30 minutes of continuous low-impact dancing (60–70% of maximum heart rate), and a 10-minute cool-down phase (low-intensity walking and static stretching of the main muscle groups). The program included traditional Colombian dances (bambuco, cumbia, porro, guabina, and torbellino), characterized by rhythmic displacements and coordinated movements, performed under weight-bearing conditions but without the inclusion of jumps, high-magnitude vertical impacts, or external loading, and was delivered by a dance instructor under standardized conditions.

Protocol adherence was defined as participants attending at least 90% of the intervention sessions while participant retention was considered acceptable if at least 85% of women remained in the study until its completion (Hakestad et al., 2015). Safety was evaluated by the absence of serious adverse events throughout the intervention period (Mesinovic et al., 2025).

### 2.5 Outcomes

As a primary outcome, bone mineral density (g·cm^-^²) at the femoral neck and lumbar spine was measured via DXA (Lunar Prodigy Advance, GE Healthcare, Chicago, IL, USA) using TBS iNsight software. Participants were instructed to avoid calcium intake 24 hours prior, wear loose clothing, and remove jewelry. For the lumbar spine measurement, participants were positioned supine with legs bent on a support to reduce lumbar lordosis and L1–L4 vertebrae were scanned. For the femoral neck measurement, the right hip was internally rotated (15–30°) with the minor trochanter excluded from view. Appropriate patient positioning was based on previous clinical recommendations to estimate BMD with DXA method (Themeli and Triantafyllopoulos, 2021). Neither a specific vertebral exclusion protocol nor a formal image adjudication process was applied. Two certified technicians (with +7 years of experience) independently analyzed scans to minimize errors. Results were compared to NHANES III reference data for T-/Z-scores considering age- and sex-specific values.

### 2.6 Sample size

This study was conceived as a pilot trial. The sample size of 20 participants (10 per group) was based on practical constraints (convenience sampling) and aligned with previous pilot studies on resistance training in older adults such as Balachandran et al. (2014) (*n* = 21) and Vargas-Molina et al. (2022) (*n* = 21). While no formal *a priori* power analysis was conducted, this sample size is typical for early-phase feasibility trials.

### 2.7 Allocation

Participants were non-randomly assigned to either the experimental or control group according to their schedule availability and predefined session arrangements. The allocation did not involve an explicit selection of the intervention type by the participants. To reduce measurement bias, outcome assessors were blinded to group assignment. Both groups received the same session frequency (three times per week) and professional supervision to enhance procedural consistency and minimize performance bias.

### 2.8 Statistical analysis

Descriptive statistics are reported as mean and standard deviation (SD) with the corresponding 95% confidence interval (95% CI). Based on current recommendations to improve data analysis practices (Martin and Teste, 2022), we employed an estimation, robust and Bayesian approach, consistent with methodologies detailed in prior studies involving older adult populations (Bonilla et al., 2021; Vargas-Molina et al., 2022; Vargas-Molina et al., 2024). To determine statistical significance, we examined the 95% CIs for the difference between the mean change scores (Δ = Week 16–Week 0). If the 95% CI excludes zero, the difference will attain significance at the *p* < 0.05 level. Effect size was calculated as unbiased Cohen’s *d* (d_unb_). Following established benchmarks, a result of ≤0.2 was considered a small, 0.5 as a moderate, and ≥0.8 as a large effect (Rosenthal, 1996). In addition, a minimal clinically important difference was defined as a change of +0.03 g·cm^-2^ for any bone section, as recommended for non-obese post-menopausal women (Briot et al., 2018; Dorilleau et al., 2024). Statistical significance, strength of Bayesian evidence, and clinical relevance were interpreted as complementary but distinct inferential constructs. Gardner-Altman estimation plots were generated to display the paired-data before and after the intervention. In addition, we applied Yuen-Dixon’s paired-sample T-test, as a robust method suitable for small sample sizes. This approach employs 20% trimmed means (μ_t_) and incorporates bootstrapping to enhance Type I error control, particularly for skewed data distributions. Finally, the Yuen test for independent groups was used for the pairwise comparisons of the Δ between groups. Findings were confirmed with frequentist and Bayesian ANCOVA adjusting for baseline values. We estimated the likelihood ratio (also called the Bayes Factor [BF]), which is the most widely accepted measure to quantify how much evidence a data set provides for a hypothesis. Statistical analyses were performed using the Wilcox’ Robust Statistics (WRS2) package and JASP module ‘jsq’ within the R statistical computing environment (R Core Team, 2013).

## 3 Results

**Figure 2:**
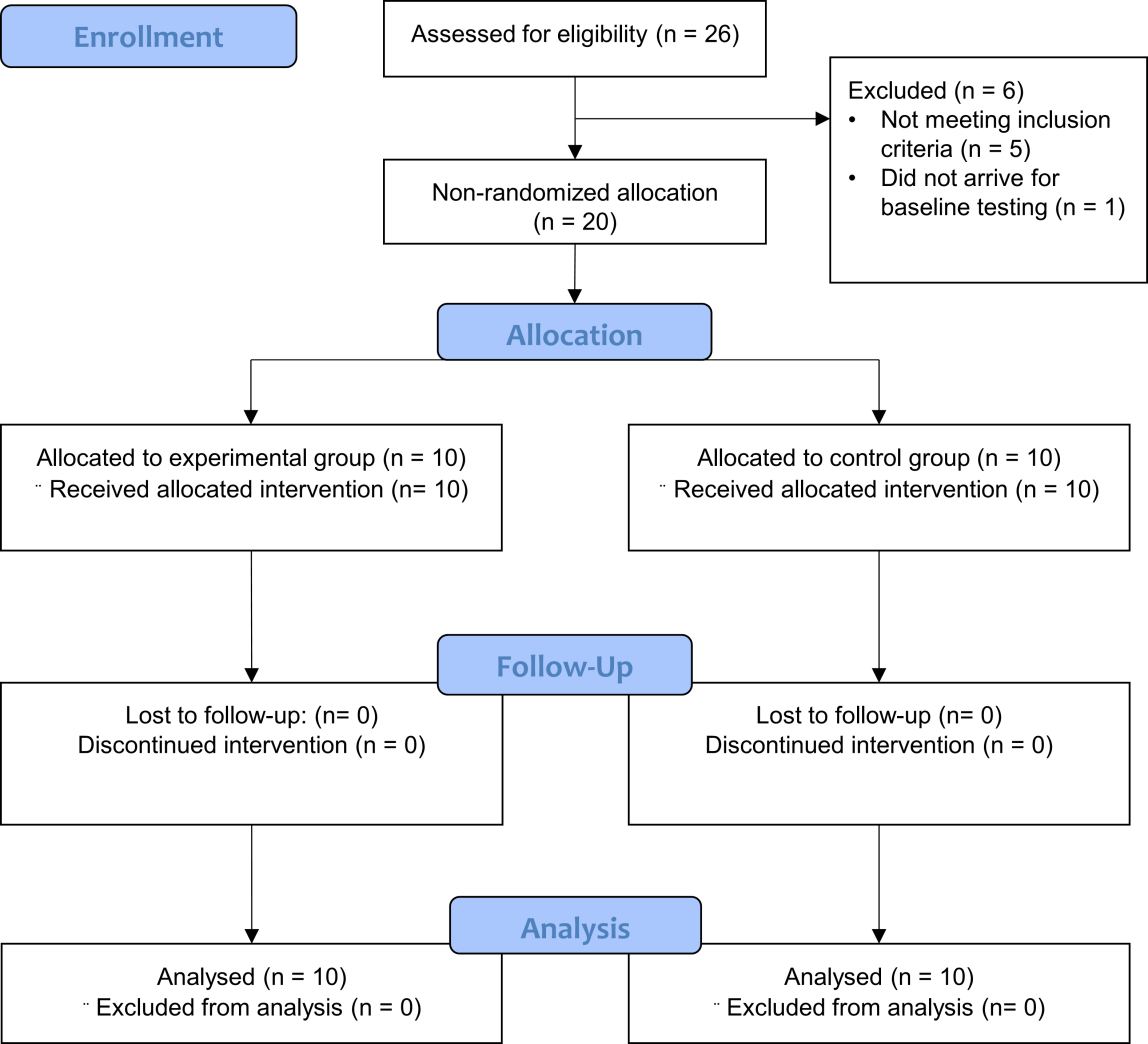
CONSORT flow diagram.

Twenty women (65.3 [3.23] years; 1.58 [0.08] m; 69.3 [12.9] kg) completed the study and were included in the analysis (**Figure 2**). Two participants from the experimental group reported localized muscle pain during the intervention period following training sessions. These cases were successfully managed through rest periods and exercise intensity modifications, with no instances of significant muscle damage identified at any point during the study. Thus, adherence reached 98% while participant retention was 100%. No serious adverse events were reported during the 16-week intervention period, confirming the safety and tolerability of the program.

A total of 26 women were assessed for eligibility. Six were excluded (five did not meet inclusion criteria and one did not attend baseline testing). Twenty participants were allocated to the study groups (*n* = 10 per group). No participants were lost to follow-up or excluded from the analysis (**Figure 2**).

The participants’ characteristics analysis revealed no statistically significant differences between the groups for any of the assessed variables (Yuen’s independent samples test, p > 0.05) at baseline, as detailed in **Table 1**.

**Table 1:** Descriptive information of the participants.

Variable	Control (*n* = 10) Mean (SD) [95% CI]	Experimental (*n* = 10) Mean (SD) [95% CI]	*P*-value
Age (years)	65.8 (3.12) [63.6, 68.0]	64.70 (3.40) [62.3, 67.1]	0.461
Stature (m)	1.55 (0.07) [1.50, 1.60]	1.60 (0.08) [1.54, 1.66]	0.077
Body mass (kg)	64.2 (6.81) [59.3, 69.1]	74.4 (15.80) [63.1, 85.7]	0.200

*p* < 0.05 (two-tailed) indicates a statistically significant difference between CON and EXP based on the Yuen-Dixon test.

### 3.1 Outcomes

**Figure 3:**
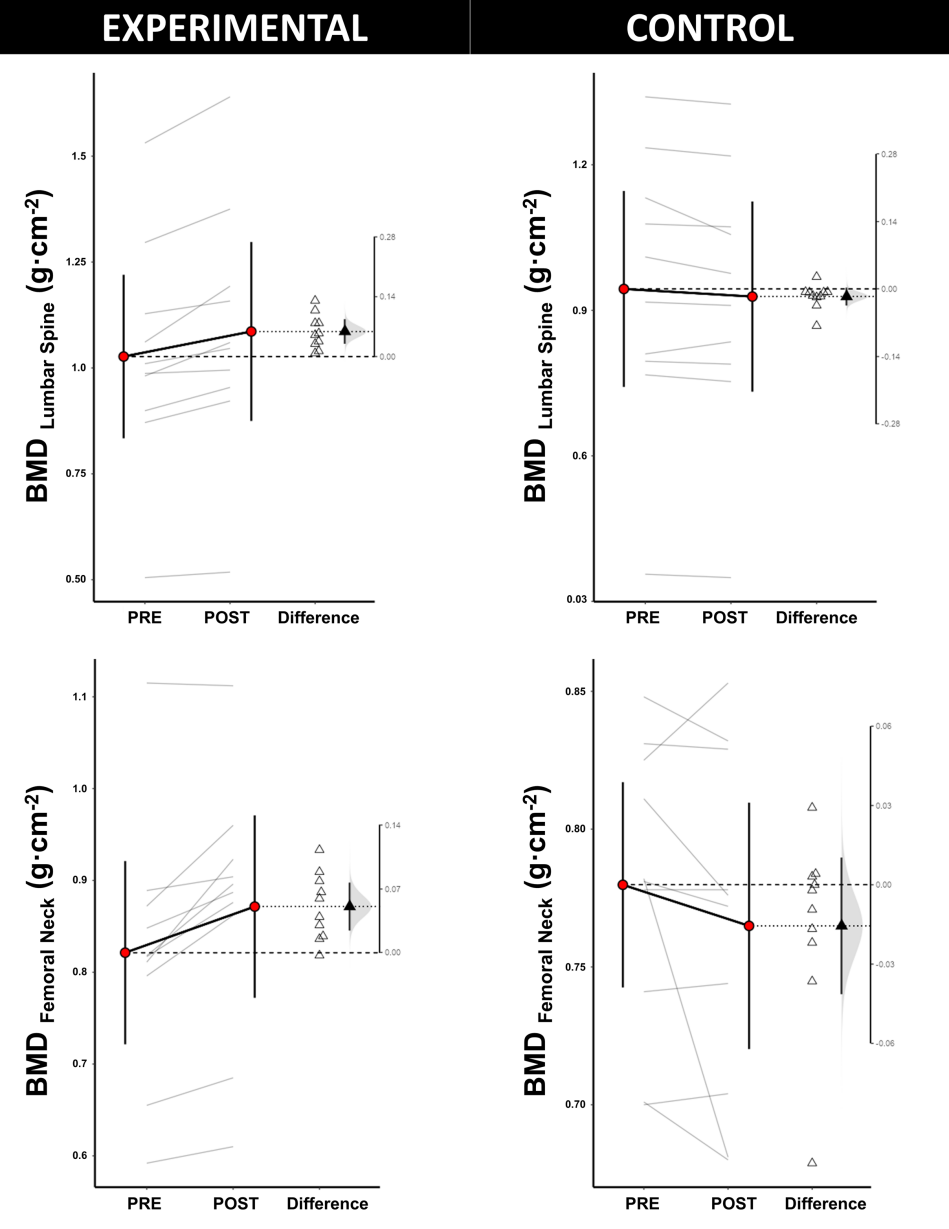
Results before and after on BMD at lumbar spine and femoral neck. Paired data are shown as lines. The difference between the initial (pre-test) and final (post-test) means is plotted on a floating difference axis, whose zero is aligned with the pre-test mean. The filled dark triangle marks the difference on that axis and the 95% CI on that difference is displayed. The differences are shown as open triangles on the difference axis. BMD, bone mineral density (g/cm²).

The results of the within-participant comparison (analysis on paired data) are expressed as Δ (SD) [95% CI]; d_unb_ [95% CI]; Yuen’s paired-test *p* value). After 16 weeks, lumbar spine (+0.05 (0.04) [0.03, 0.08] g·cm^-2^; 0.20 [0.12, 0.33]; *p* = 0.026) and femoral neck (+0.05 (0.03) [0.02, 0.07] g·cm^-2^; 0.35 [0.18, 0.60]; *p* = 0.003) BMD had a statistically significant increase in the EXP group. No significant pre-post differences were observed in the CON group (**Figure 3**). Robust paired samples t-tests confirmed significant intervention-related changes exclusively in the EXP group (see **Table 2**).

**Table 2:** Pre- and post-intervention data on the main study variables.

Variable	Group	Week 0 $\bar{x}$ (SD)	Week 16 $\bar{x}$ (SD)	Δ (SD) [95% CI]	d_unb_ [95% CI]	*P* value
BMD _Lumbar Spine_ (g·cm^-2^)	EXP	1.02 (0.26)	1.08 (0.29)	0.05 (0.04) [0.03, 0.08] *	0.20 [0.12, 0.33]	0.026
	CON	0.94 (0.28)	0.92 (0.27)	–0.01 (0.02) [–0.03, 0.002]	–0.05 [–0.12, 0.00]	0.372
BMD _Femoral Neck_ (g·cm^-2^)	EXP	0.82 (0.13)	0.87 (0.13)	0.05 (0.03) [0.02, 0.07] *	0.35 [0.18, 0.60]	0.003
	CON	0.77 (0.05)	0.76 (0.06)	–0.01 (0.03) [–0.03, 0.009]	–0.25 [–0.68, 0.11]	0.262

Data is presented as mean ( $\bar{x}$) and standard deviation (SD). Δ, mean change; BMD, bone mineral density; CI, confidence interval; CON, control group; EXP, experimental group; d_unb_, unbiased Cohen’s d (also known as Hedge’s g). * Statistically significant change (*p* < 0.05).

Robust pairwise comparisons of intergroup Δ values (*Yuen’s independent samples test*) revealed statistically significant differences between EXP and CON groups for both lumbar spine BMD (*t* = 5.22, *p* = 0.003) and femoral neck (*t* = 3.58, *p* = 0.01), with the EXP group showing clinically superior outcomes. A Bayesian ANCOVA adjusting for baseline values provided strong evidence supporting the treatment effects observed in the EXP group. For lumbar spine BMD, the model combining group and baseline values showed overwhelming support (BF_m_ = 7.00×10¹³, P(M|data) = 0.987), with a 98.7% posterior probability that group membership influenced outcomes (BF_in_c_1_ = 76.6). Similarly for femoral neck BMD, the combined model was strongly favored (BF_m_ = 1.07×10^7^, P(M|data) = 0.964), with 96.4% posterior inclusion probability for group effects (BF_in_c_1_ = 26.6). Although a numerical between-group difference in baseline body mass was observed (EXP: 74.4 [15.80] kg vs. CON: 64.2 [6.81] kg), it did not reach statistical significance (*p* = 0.200). More importantly, a Bayesian ANCOVA confirmed that including body mass as a covariate did not meaningfully change the evidence supporting the intervention effect, either for lumbar spine (BF Inclusion for Group = 68.4) or femoral neck (BF Inclusion for Group = 26.4). In fact, body mass itself showed evidence against a relevant confounding effect (BF Inclusion = 0.048 and 0.129, respectively), reinforcing that the observed BMD improvements are attributable to the exercise intervention rather than baseline weight differences.

These results converged with the frequentist ANCOVA showing large treatment effects favoring the EXP group in both lumbar spine (adjusted mean difference = +0.073 units, *p* < 0.001, η²p=0.565) and femoral neck (adjusted mean difference = +0.0665 units, *p* < 0.001, η²p=0.485). Thus, the robust delta analyses (Yuen’s test) and both Bayesian and frequentist approaches consistently indicated that the control group exhibited poorer outcomes.

## 4 Discussion

This pilot study indicates that a 16-week progressive multimodal resistance training program with varied muscle actions and ROM may improve BMD in older women (60–70 years) with osteopenia. The intervention group showed substantial BMD improvements (>0.03 g·cm^-2^) at both lumbar spine and femoral neck sites compared to controls, supporting the potential efficacy of this novel training approach. When compared with previous exercise trials in postmenopausal women with osteopenia, the magnitude of BMD gains observed in our study is comparable to or slightly greater than those reported in meta-analyses of resistance training interventions, which typically show weighted mean differences ranging from +0.01 to +0.03 g·cm^-^² at the lumbar spine (Kelley et al., 2001). These findings align with recent systematic reviews confirming resistance training’s osteogenic potential in older adults (Borba-Pinheiro et al., 2016; Mohebbi et al., 2023), while extending current knowledge through innovative intensity control via ROM and muscle action periodization.

The American College of Sports Medicine (ACSM) advocates the FITT-P system (frequency, intensity, time, type, progression) for this demographic, combining strength/balance exercises with cardiorespiratory training targeting core, quadriceps, hamstrings, and gluteal muscles (Nelson et al., 2007). Optimal protocols suggest gradual intensity progression (50-80% 1RM), 2–3 weekly sessions, and 48-hour recovery periods. The 98% adherence rate further supports the clinical feasibility of this approach, achieved through professional supervision and individualized progression - key factors emphasized in the recent international consensus of exercise for older adults (Izquierdo et al., 2021).

Our methodology offers distinct advantages over conventional high-load approaches. By controlling intensity through ROM and muscle action parameters rather than absolute loads, the program minimized injury risk while maintaining efficacy - an important consideration for older adults with mobility limitations (Ciolac and Rodrigues-da-Silva, 2016). This aligns with emerging evidence that submaximal loads can effectively improve BMD when appropriately periodized (Banitalebi et al., 2021). Our ROM-based periodization approach achieved BMD improvements similar to those reported in high-load resistance training studies (e.g., 70–85% 1RM) without requiring maximal or near-maximal loads, which is particularly relevant for older adults with joint concerns or low baseline fitness (Fragala et al., 2019). The inclusion of functional movements also addresses calls for more ecologically valid exercise prescriptions in osteoporosis prevention (Borba-Pinheiro et al., 2016). Notably, lumbar spine BMD improvements exceeded femoral neck gains, consistent with trabecular bone’s greater metabolic activity and responsiveness to mechanical stimuli compared to cortical-rich sites (Watson et al., 2018). This site-specific response pattern mirrors findings from previous clinical research (Holubiac et al., 2022) and a meta-analysis of exercise interventions in postmenopausal women (Massini et al., 2022), reinforcing the biological plausibility of our results.

The program’s success can be explained from multiple synergistic mechanisms. First, the combination of multi-joint exercises with progressive ROM variations created optimal osteogenic loading patterns. In addition, Kemmler and von Stengel (2014) have shown that increased exercise frequency correlated with greater BMD improvements in postmenopausal women with osteopenia. Resistance training has shown to generate bone health benefits through mechanical stress, stimulation of osteoblast activity, suppression of osteoclast function, and promotion of bone formation through Wnt/β-catenin pathways (Hong and Kim, 2018; Carina et al., 2020). In contrast, the control group (dance therapy) showed minimal or no BMD changes, which aligns with previous evidence indicating that low-impact weight-bearing activities without progressive overload are insufficient to counteract age-related bone loss in osteopenic women (Guadalupe-Grau et al., 2009). Second, the periodized integration of concentric, eccentric, and isometric actions provided diverse mechanical stimuli, with eccentric phases particularly effective in inducing bone strain (Singh et al., 2023). This multi-faceted approach corroborates recent recommendations for exercise prescription in postmenopausal osteopenia/osteoporosis (Mohebbi et al., 2023), while addressing a critical gap in current protocols by indicating the potential ROM’s utility as a progression variable.

The increase in BMD might translate into a reduced risk of fractures, making it a crucial goal in osteoporosis prevention. Furthermore, the accessibility, adaptability, and safety of this program for older women can facilitate its adoption and long-term sustainability, suggesting its implementation in different settings, ranging from fitness centers to clinical rehabilitation facilities. Although this pilot study was not powered to detect efficacy outcomes, the observed effects on BMD, combined with excellent adherence (98%), complete retention (100%), and absence of serious adverse events, provide sufficient feasibility evidence to justify a future fully powered randomized controlled trial. These feasibility metrics met pre-established thresholds (≥90% adherence, ≥85% retention, and safety), supporting progression to a definitive trial.

### 4.1 Strengths and limitations

The significant improvements in BMD observed in this study suggest that periodizing muscle action types and ROM represents an effective strategy for designing future rehabilitation and osteoporosis prevention programs. The involvement and supervision of qualified professionals ensured the program’s adherence (98%) and safety. This not only allowed participants to perform the exercises correctly but also provided continuous support and guidance, contributing to statistically significant outcomes. In fact, the use of robust statistical methods (including 20% trimmed means) minimized the influence of potential outliers and extreme responses, which is particularly important given the small sample size. Future studies should investigate optimal dosing parameters (frequency/volume) and long-term effects, while incorporating advanced imaging to assess microarchitectural changes.

Several limitations warrant consideration. The single-region sampling (Colombian women) and small sample size constrain generalizability, though this is partially mitigated by the robust statistical methods implemented. The pre-post design without middle assessments limits our ability to identify critical windows for BMD adaptation, a limitation noted in similar trials (Holubiac et al., 2022). No vertebral exclusion or formal adjudication protocol was applied to lumbar spine vertebrae with age-related changes. Additionally, the 16-week duration, although sufficient to identify preliminary changes in BMD, may be considered limited given the slow nature of bone adaptation, which restricts the assessment of long-term effects. However, this duration is consistent with the pilot nature of the study, whose purpose was to explore preliminary signals of effect, as well as to evaluate the feasibility, safety, and adherence of the intervention (Liao et al., 2021; Liu et al., 2023). Furthermore, habitual physical activity outside the intervention was not quantified using objective instruments, which could represent a potential confounding factor, particularly in the context of a non-randomized study design.

## 5 Conclusion

A 16-week progressive multimodal resistance training program with varied muscle actions and ROM can significantly improve BMD in older women with osteopenia. The adaptability and safety of this approach further indicate its potential for widespread implementation in both clinical and community settings. This novel methodology offers a structured and controlled approach to regulating training intensity and progression, suggesting efficacy as both a safe and effective intervention for improving bone health parameters. Importantly, the high adherence, full retention, and absence of serious adverse events confirm the feasibility of the intervention and support the design of a future fully powered randomized controlled trial.

## Data Availability

The raw data supporting the conclusions of this article will be made available by the authors, without undue reservation.
