# Supplementary material for: Effects of progressive multimodal resistance training with varied muscle actions and range of motion on bone mineral density in osteopenic older women: a pilot study
Source: Front Physiol. 2026 Jul 6;17:1811518. doi: 10.3389/fphys.2026.1811518 (PMC13383042; doi:10.3389/fphys.2026.1811518)
Supplement: Supplementary file 1 [file Table1.docx]

Table S1. Detailed 16-week progression of the multimodal resistance training program

| **Phase (Muscle action)** | **Phase 1 - Concentric action** | | | | **Phase 2 - Concentric-isometric action** | | | | **Phase 3 - Concentric-eccentric action** | | | | **Phase 4 - Concentric-eccentric action** | | | |
| --- | --- | --- | --- | --- | --- | --- | --- | --- | --- | --- | --- | --- | --- | --- | --- | --- |
| Month | May | | | | June | | | | July | | | | August | | | |
| Week | 1 | 2 | 3 | 4 | 5 | 6 | 7 | 8 | 9 | 10 | 11 | 12 | 13 | 14 | 15 | 16 |
| Start and end dates | 2–7 May | 9–13 May | 16–20  May | 23–27 Mayo | 30 -3 June | 6–10 June | 13–17  June | 20–24 June | 27–1 July | 4–8 July | 11–15 July | 18–22 July | 25-29 July | 1–5 August | 8–12 August | 15-19 August |
| Sessions per week | 3 | 3 | 3 | 3 | 3 | 3 | 3 | 3 | 3 | 3 | 3 | 3 | 3 | 3 | 3 | 3 |
| Average session volume (min) | 45’ | 45’ | 45’ | 45’ | 50’ | 50’ | 50’ | 45’ | 50’ | 50’ | 50’ | 45’ | 55’ | 55’ | 55’ | 45’ |
| Total weekly volume (min) | 135 | 135 | 135 | 135 | 150 | 150 | 150 | 135 | 150 | 150 | 150 | 135 | 165 | 165 | 165 | 135 |
| Warm-up (5’) and cool down (10’) | 45’ | 45’ | 45’ | 45’ | 45’ | 45’ | 45’ | 45’ | 45’ | 45’ | 45’ | 45’ | 45’ | 45’ | 45’ | 45’ |
| Functional exercises with light loads in low-complexity domestic work activities (ses/min) | 3/21 (7’) | 3/21 (7’) | 3/21 (7’) | 3/21 (7’) | 1/8 (8’) | 1/8 (8’) | 1/8 (8’) | 1/6 (6’) |  |  |  |  |  |  |  |  |
| Functional exercises with light loads in moderate-complexity domestic work activities (ses/min) |  |  |  |  | 2/16 (8’) | 2/16(8’) | 2/16 (8’) | 2/14(7’) | 3/24(8’) | 3/24 (8’) | 3/24 (8’) | 3/21 (7’) | 3/27 (9’) | 3/27 (9’) | 3/27 (9’) | 3/21’(7’) |
| Multi-joint exercises on machines under passive stability conditions |  |  |  |  |  |  |  |  |  |  |  |  |  |  |  |  |
| Individual exercises (horizontal method) | 69’ (23) | 69’ (23) | 30’  (10) | 30’  (10’) | 81’  (27) | 36’  (12) | 39’  (13) | 30’  (10) |  |  |  |  |  |  |  |  |
| Number of exercises/series/repetitions | 5/2  6-8 | 6/3  6-8 | 3/2  8-10 | 2/2  8-10 | 6/2  4-6 | 3/2  4-6 | 3/2  4-6 | 2/2  4-5 | 6/2  4-6 | 3/2  4-6 | 3/2  4-6 | 2/2  4-6 | 6/2  4-6 | 3/2  6-8 | 3/2  6-8 | 2/2  4-6 |
| Range of Motion Level | Severely Limited Range of Motion | | Limited Range of Motion | | Limited Range of Motion | | | | Moderate Range of Motion | | | | Moderate Range of Motion | | | |
| Range of Motion in Degrees Tibiofemoral Joint (TFJ) and Acetabulofemoral Joint (AFJ) | 120° -110° TFJ. 125°-100 AFJ | | 109º - 100º - TFJ. 99º- 80º AFJ | | 109º - 100º - TFJ.  99º- 80º AFJ | | | | 99°- 90° TFJ.  79°-60° AFJ | | | | 99°- 90° TFJ  79°-60° AFJ | | | |
| Execution speed (seconds) / Rest interval between series (min) | 2/1 | 2 /1.0 | 2/1.0 |  | 2-2/  1.5 | 2-3/  1.5 | 2-3/  1.5 | 2-2/  1.5 | 2-2/  1.5 | 2-2/  1.5 | 2-2/  1.5 | 2-2/  1.5 | 2-4/  2.0 | 2-4/  2.0 | 2-4/  2.0 | 2-2/  2.0 |
| Double series exercises (vertical method) |  |  | 39’  (13) | 39’  (13’) |  | 45’  (15) | 45’  (15) | 39’  (13) |  |  |  |  |  |  |  |  |
| Number of exercises/double series/repetitions |  |  | 4/2  6-8 | 3/2  6-8 |  | 4/2  4-6 | 4/2  4-6 | 4/2  4-5 |  | 4/2  6-8 | 4/2  6-8 | 4/1  6-8 |  |  |  |  |
| Range of motion |  |  | 2 | 2 |  | 3 | 3 | 3 |  | 3 | 3 | 3 |  |  |  |  |
| Execution speed (seconds) / Rest interval between double series (min) |  |  | 2/2 | 2/2 |  | 2-3/  3.0 | 2-3/  3.0 | 2-2/  3.0 |  | 1-1/  3.0 | 1-1/  3.0 | 1-1/  3.0 |  |  |  |  |
| Triple series exercises (vertical method) |  |  |  |  |  |  |  |  |  |  |  |  |  |  |  |  |
| Number of exercises/triple series/repetitions |  |  |  |  |  |  |  |  |  |  |  |  |  | 6/2  6-8 | 6/2  6-8 | 6/1  6-8 |
| Range of motion |  |  |  |  |  |  |  |  |  |  |  |  |  | 3 | 3 | 3 |
| Execution speed (seconds) / Rest interval (between exercises - between triple series) (min) |  |  |  |  |  |  |  |  |  |  |  |  |  | 1-2/  1-4 | 1-2/  1- 4 | 1-2/  1-4 |
